# Comparison of Hydrogen Embrittlement Susceptibility of Different Types of Advanced High-Strength Steels

**DOI:** 10.3390/ma15093406

**Published:** 2022-05-09

**Authors:** Sangwon Cho, Geon-Il Kim, Sang-Jin Ko, Jin-Seok Yoo, Yeon-Seung Jung, Yun-Ha Yoo, Jung-Gu Kim

**Affiliations:** 1 School of Advanced Materials Science and Engineering, Sungkyunkwan University (SKKU), Suwon 16419, Korea; jsw2811@gmail.com (S.C.); geonil1027@g.skku.edu (G.-I.K.); tkdwls121@skku.edu (S.-J.K.); wlstjr5619@skku.edu (J.-S.Y.); 2 Steel Solution Research Laboratory, POSCO Global R&D Center, Incheon 21985, Korea; ysj799@posco.com (Y.-S.J.); yunha778@posco.com (Y.-H.Y.)

**Keywords:** advanced high-strength steel, hydrogen embrittlement, hydrogen trapping, thermal desorption spectroscopy

## Abstract

This study investigated the hydrogen embrittlement (HE) characteristics of advanced high-strength steels (AHSSs). Two different types of AHSSs with a tensile strength of 1.2 GPa were investigated. Slow strain rate tests (SSRTs) were performed under various applied potentials (E_app_) to identify the mechanism with the greatest effect on the embrittlement of the specimens. The SSRT results revealed that, as the E_app_ increased, the elongation tended to increase, even when a potential exceeding the corrosion potential was applied. Both types of AHSSs exhibited embrittled fracture behavior that was dominated by HE. The fractured SSRT specimens were subjected to a thermal desorption spectroscopy analysis, revealing that diffusible hydrogen was trapped mainly at the grain boundaries and dislocations (i.e., reversible hydrogen-trapping sites). The micro-analysis results revealed that the poor HE resistance of the specimens was attributed to the more reversible hydrogen-trapping sites.

## 1. Introduction

The need to reduce environmental harm is a growing global concern. Accordingly, the automotive industry is striving to improve fuel efficiency and reduce carbon dioxide emissions to protect the environment. Globally, the industry is pushing for fuel efficiency improvements via two routes: high-efficiency engines and lightweight body designs [1,2,3]. A vehicle’s body weight accounts for 40% of its fuel efficiency factors; therefore, reducing this weight has the greatest impact on improving fuel efficiency. Generally, a 100-kg reduction in body weight lowers carbon dioxide emissions by 7.5 to 12.5 g/km, significantly enhancing the fuel efficiency. To reduce the weight of cars, manufacturers may use nonferrous materials (e.g., resin, aluminum alloy, and magnesium alloy) [4,5,6,7,8]; specific methods (e.g., the miniaturization of parts); or different types of high-strength steels [9,10,11]. Although nonferrous materials used in automotive structures such as aluminum alloy and magnesium alloy are lighter than steel, they are also weaker, and their thicknesses must be increased to maintain body stiffness. Additionally, lightweight materials must be used in combination with other materials, such as carbon fiber-reinforced plastic, to maintain the required body stiffness. Therefore, research and development into various types of advanced high-strength steels (AHSSs) are currently underway.

Generally, the mechanical properties of steel are enhanced using methods such as solid solution, grain refinement, or precipitation; however, in the case of AHSS, phase transformation-based methods are also used. Enhancing the mechanical properties of an AHSS increases its corrosivity and sensitivity to a delayed fracture, i.e., stress–corrosion cracking (SCC) and hydrogen embrittlement (HE), which are the main problems associated with AHSS [12].

Over the past decades, a lot of research on HE in AHSSs has been conducted: effects of the strength, microstructure, and types of defects of AHSSs. V. G. Khanzhin et al. [13,14] studied the influence of precipitate and mechanical properties on HE. According to the studies, the higher density of precipitates in a structure, the lower HE resistance, since the secondary phase particles influence both the stage of initiating hydrogen cracks and the crack growth kinetics to a critical value. Additionally, mechanical properties, their strength and toughness, affects the nucleation of hydrogen cracks, possibility of their propagation, and the kinetics of growth to a critical size.

In AHSS, the delayed fracture phenomenon is caused mainly by HE. Hydrogen inside the steel is preferentially trapped in lattice defects, such as voids, dislocations, and grain boundaries, as well as in various carbides and precipitates [15,16]. Additionally, after entrapment, hydrogen is concentrated in certain areas by stress, leading to the propagation of internal cracks and, eventually, to delayed fractures. However, the exact cause and mechanism of the delayed fracture phenomenon have not been identified to date. This is because, in addition to HE, a delayed fracture can result from the combined effects of other variables, including the stresses acting on the steel, the microstructures, mechanical properties, surface conditions, and internal cracks. Further research is required to determine the exact cause of HE. Therefore, this study uses slow strain rate tests (SSRTs), a microstructural analysis, and a thermal desorption spectroscopy (TDS) analysis to investigate the HE mechanisms of two different types of AHSSs with the tensile strength of 1.2 GPa.

## 2. Materials and Methods

**Figure 1:**
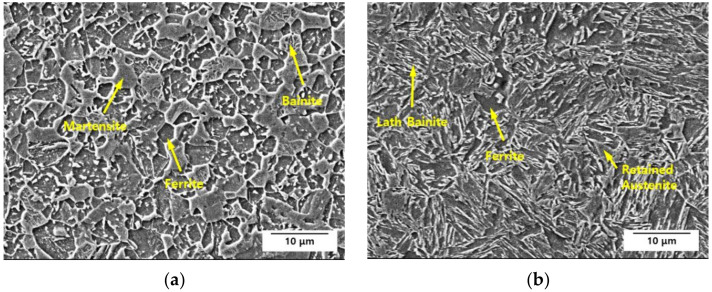
Scanning electron microscopy images of the microstructure of (**a**) Steel A and (**b**) Steel B etched with a 2% nital solution.

**Figure 2:**
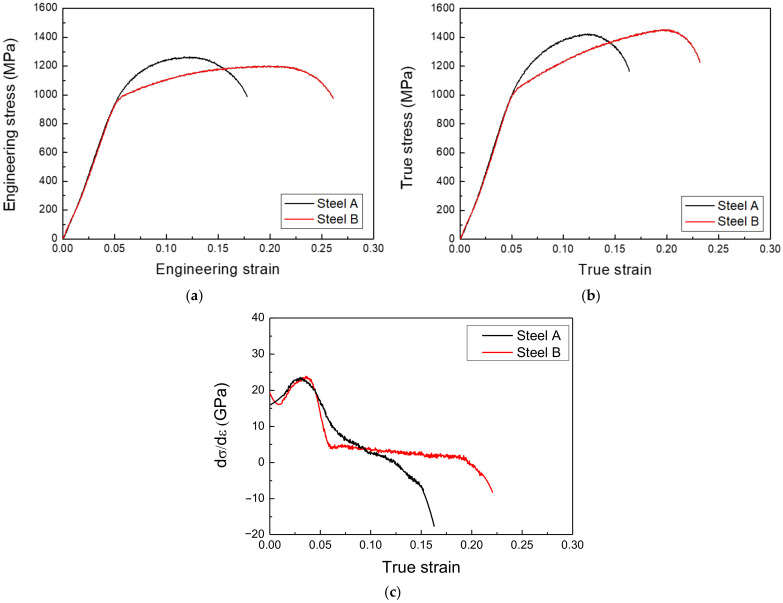
(**a**) Engineering stress–strain curves, (**b**) true stress–strain curves, and (**c**) strain-hardening rate vs. true strain curves for Steel A and Steel B obtained by tensile tests.

This study used two different types of AHSSs with a tensile strength of 1.2 GPa. Table 1 provides their chemical compositions. Figure 1 presents the specimens’ microstructural images, and Figure 2 shows the mechanical properties obtained by the tensile test. Steel A was comprised of fine grains with complex phases of ferrite, bainite, martensite, and a small fraction of retained austenite, with Ti and/or Nb precipitates for enhancing the tensile strength and ductility. Since this steel was cooled slowly after soaking in the austenite region, its main phases were bainite and martensite. Steel B was also a multiphase AHSS comprising ferrite, bainite, a relatively higher fraction of retained austenite, and a small portion of martensite. Under an applied stress, the phase transformation of the retained austenite increased the ductility of Steel B. As shown in Figure 2c, Steel B showed a uniform strain-hardening rate range, which is evidence of a transformation-induced plasticity effect.

### 2.1. Electrochemical Tests

**Figure 3:**
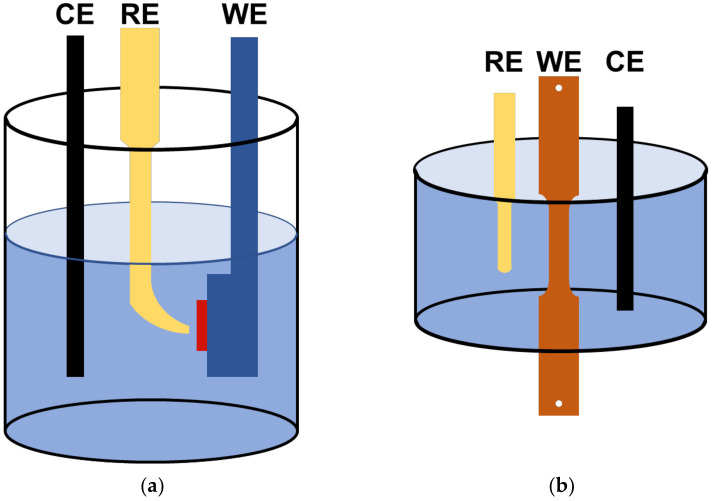
Schematic diagram of the three-electrode cell configuration used in (**a**) potentiodynamic polarization test and (**b**) SSRTs. CE, RE, and WE refer to counter electrode, reference electrode, and working electrode, respectively.

Potentiodynamic polarization test was performed to analyze the corrosion behavior of AHSSs and determine the applied potentials in the SSRTs. For the electrochemical tests, the specimens were cut into dimensions of 1.5 × 1.5 cm^2^, abraded up to #600 with silicon–carbide paper, degreased with ethanol, and dried with N_2_ gas. The electrochemical test environment used a modified Society of Automotive Engineers’ (M-SAE) solution at 25 °C (room temperature) (see Table 2 for the chemical compositions). All the electrochemical experiments were performed with a triple-electrode electrochemical cell, as shown in Figure 3. The counter electrode was a graphite rod, and the reference electrode was a saturated calomel electrode (SCE). The open-circuit potential (OCP) was established over 3 h. Potentiodynamic polarization tests were conducted with a potential sweep of 0.166 mV/s in accordance with ASTM G5. After the samples were stabilized in an M-SAE solution at room temperature for 1 h, SSRTs were conducted under applied potentials (E_app_) of −600, −750, and −1500 mV_SCE_ based on the potentiodynamic polarization test.

### 2.2. Slow Strain Rate Tests

**Figure 4:**
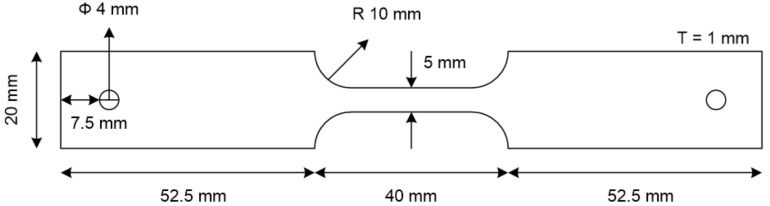
Dimensions of the specimen used in slow strain rate tests.

A schematic image of the specimen for the SSRTs is presented in Figure 4. First, the critical strain rate was determined by various SSRTs in the OCP state; then, under the E_app_ values listed in Section 2.1, SSRTs were conducted at a strain rate of 10^−5^. After the tests, each fractured specimen was cleaned with ethanol and transferred into liquid nitrogen as soon as possible. Then, the fracture surface was observed via scanning electron microscopy (SEM), and the hydrogen desorption rate was determined by a TDS analysis of the hydrogen content charged into the specimen. To enable the TDS analysis of the hydrogen content, the specimen was cut up to 10 mm from the fracture surface. To calculate the activation energy for hydrogen de-trapping, heating rates of 2 °C/min and 4 °C/min were used.

### 2.3. Analyses for Hydrogen-Trapping Sites

The grain boundary areas and austenite phase fractions of the samples were measured using electron backscattered diffraction (EBSD). X-ray diffraction (XRD) was performed at a scan rate of 1°/min, and the dislocation density was calculated using the full width at half-maximum (FWHM). Electron probe microanalysis (EPMA) and transmission electron microscopy (TEM) were used to analyze the type and characteristics of the precipitates. Before EPMA, each specimen was etched slightly with a 2% nital solution for 5 s.

## 3. Results and Discussion

### 3.1. Potentiodynamic Polarization Test

**Figure 5:**
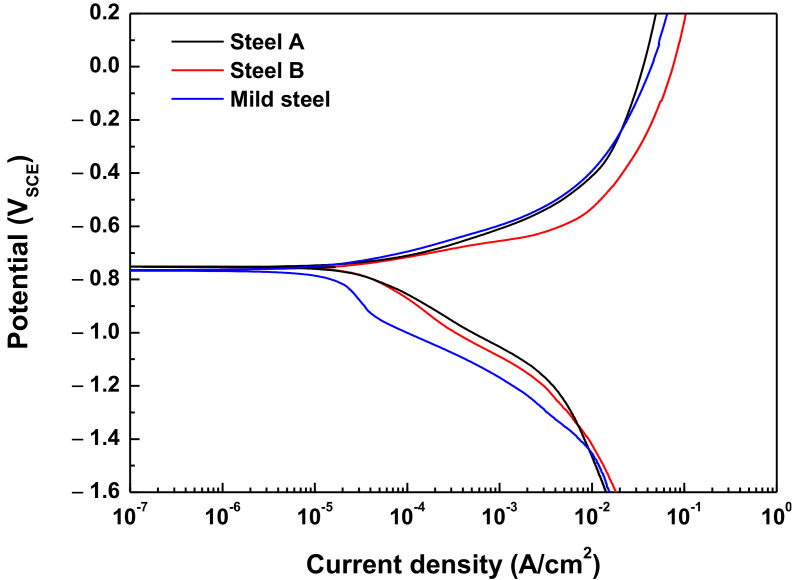
Potentiodynamic polarization curves in a modified Society of Automotive Engineers’ solution.

This study conducted potentiodynamic polarization tests to analyze the elec-tro-chemical properties of the specimens; the results are presented in Figure 5 and Table 3. Mild steel with a tensile strength of 270 MPa was used as a comparison material for the AHSS. The AHSSs exhibited higher corrosion rates than the mild steel. The anodic polar-ization curves of the AHSSs and the mild steel material were similar in shape; however, the ca-thodic polarization curve of the AHSSs shifted more to the right compared with the mild steel. This was because hydrogen evolution reactions are more likely in AHSS than in mild steel due to the higher levels of precipitates, carbides, and grain boundary densi-ties. Additionally, there are more phase types in AHSS compared with mild steel, result-ing in higher corrosion rates due to the large micro-galvanic effect between phases [17].

The electrochemical properties of both AHSSs, e.g., corrosion potential and corrosion current density, were almost identical. From the polarization curve of Steel A, the redox reaction of hydrogen (Equation (1)) produced a higher hydrogen equilibrium potential (E^0^_H_2___O/H_2__, −0.672 V_SCE_) than corrosion potential (E_corr_, −0.728 V_SCE_). Therefore, hydrogen was also generated at the corrosion potential.

(1) $$2H_{2}O+2e^{-}\to H_{2}+2{\mathrm{OH}}^{-},{\;E}_{H_{2}O/H_{2}}^{0}=-0.672V_{\mathrm{SCE}}.\;$$

### 3.2. Slow Strain Rate Tests

**Figure 6:**
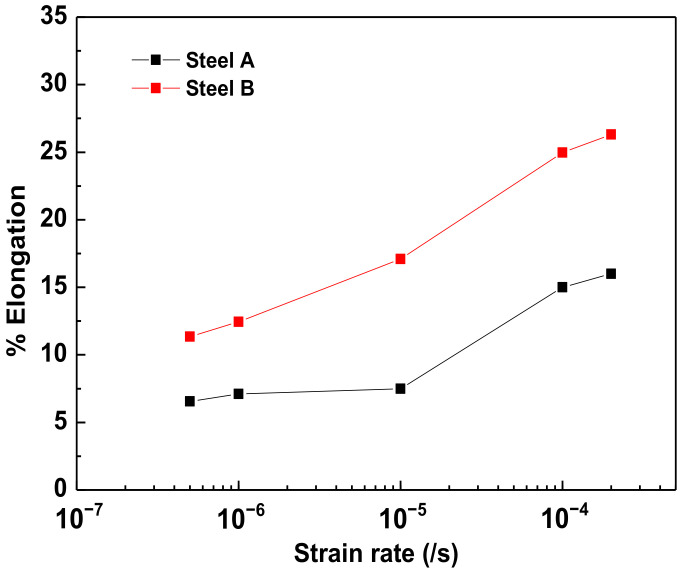
Elongation vs. strain rate in a modified Society of Automotive Engineers’ solution under an open-circuit potential state.

During an SSRT, the applied strain rate will cause differences in the occurrence of SCC and HE behaviors [18]. When the strain rate is too high, there is insufficient time for SCC to occur, resulting in only a tensile rupture. Conversely, at a low strain rate, the re-passivation of the film before the propagation of a crack by anodic dissolution means that SCC does not occur. However, HE does not require the breakdown of the passive film; instead, it is caused by hydrogen trapping inside the steel. Therefore, the lower strain rate requires more time for the hydrogen to be entrapped in the steel, making it more susceptible to HE. Accordingly, when conducting SSRTs to determine the HE characteristics, an optimal strain rate should be applied in consideration of the hydrogen trapping time. To determine the critical strain rate, the SSRTs were performed at strain rates of 2 × 10^−4^/s, 10^−4^/s, 10^−5^/s, 10^−6^/s, and 5 × 10^−7^/s in M-SAE solution under an OCP state (see Figure 6 for the results). When the strain rate was 10^−5^/s, the SSRT results revealed a relatively low elongation for both AHSSs. Therefore, the final SSRTs were performed under a strain rate of 10^−5^/s.

Both specimens showed a decreasing elongation with a decreasing strain rate, which is representative of a typical HE elongation–strain rate curve, in which a ductility minimum is not expected [18]. A low strain rate provides sufficient time for lattice diffusion, which allows hydrogen to easily enter the trapping sites.

**Figure 7:**
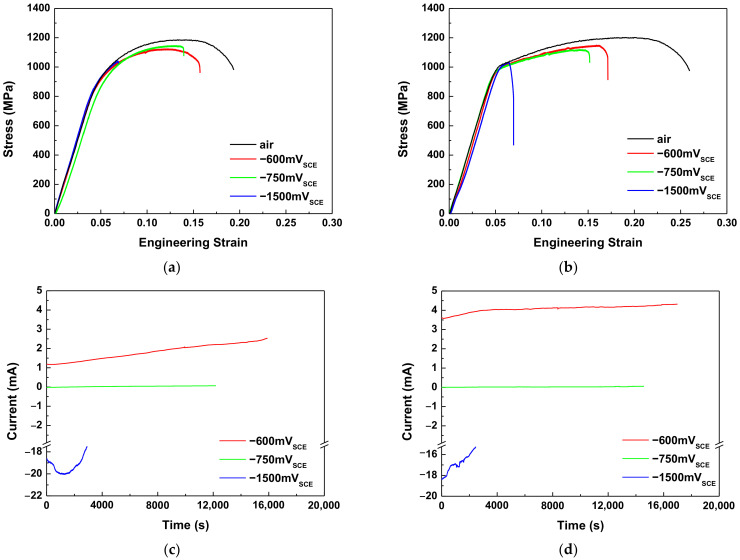
Stress–strain curves for (**a**) Steel A and (**b**) Steel B, and the current–time curves of (**c**) Steel A and (**d**) Steel B obtained during slow strain rate tests.

To minimize the hydrogen generation reactions, the applied anodic potential should be higher than the hydrogen reduction potential. Accordingly, as is shown in Figure 7 and Table 4, the SSRTs in this study were conducted using various E_app_ values. The amounts of hydrogen evolution for the E_app_ values of −1500 mV_SCE_ were calculated by integrating the base area of the current–time curves (Figure 7c,d). Since the current obtained with an E_app_ above −750 mV_SCE_ was caused by corrosion, the hydrogen evolution amounts for E_app_ values of −750 mV_SCE_ were calculated using Faraday’s law as follows [18]:

(2) $$m=\frac{I_{red,H_{2}O/H_{2}}\times t\times a}{n\times F}$$

where *m* is the reaction mass (hydrogen evolution amount, in grams), *I_red_* is the current of the reduction reaction at each E_app_ (A), *t* is the time to fracture (s), *a* is the atomic weight (g/mol), *n* is the number of electrons (*n* = 2 for Equation (1)), and *F* is the Faraday constant (96,500 C/mol).

For both AHSSs, the lower E_app_ was found to be correlated with a reduced elongation. There was an increase in the amount of corrosion with a higher anodic overvoltage, while the hydrogen evolution amount increased with the increasing cathodic overvoltage. A slightly higher amount of hydrogen was generated on Steel A compared to on Steel B.

The HE sensitivity index (I_HE_) indicates the ductility loss of the AHSSs according to the E_app_. Since the ductility loss of AHSSs with cathodic applied potential is related to HE, the I_HE_ was used to compare the HE resistance. The I_HE_ can be calculated using Equation (3), in which a higher I_HE_ is associated with increased HE sensitivities. The I_HE_ of Steel B was approximately 10% higher than that of Steel A. Therefore, compared with Steel B, Steel A had a superior HE resistance.

(3) $$I_{\mathrm{HE}}=\frac{\varepsilon_{\mathrm{air}}-\varepsilon_{\mathrm{soln}.}}{\varepsilon_{\mathrm{air}}}\times 100$$

where I_HE_ is the HE sensitivity index (%), ε_air_ is the elongation tested in air, and ε_soln._ is the elongation tested under an E_app_.

### 3.3. Fractography

**Figure 8:**
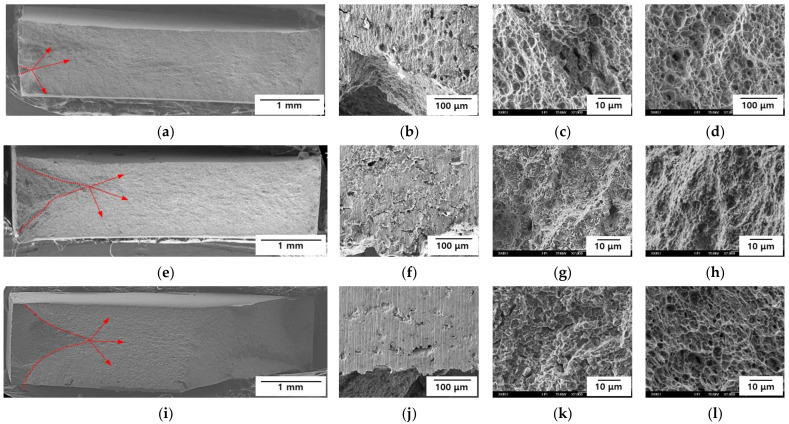
Fractography of Steel A at (**a**–**d**) −600 mV_SCE,_ (**e**–**h**) −750 mV_SCE_, and (**i**–**l**) −1500 mV_SCE_. (**a**,**e**,**i**) Entire sample, (**b**,**f**,**j**) side view, (**c**,**g**,**k**) crack initiation site, and (**d**,**h**,**l**) center. Red arrows indicate the initiation of cracks and direction of propagation.

**Figure 9:**
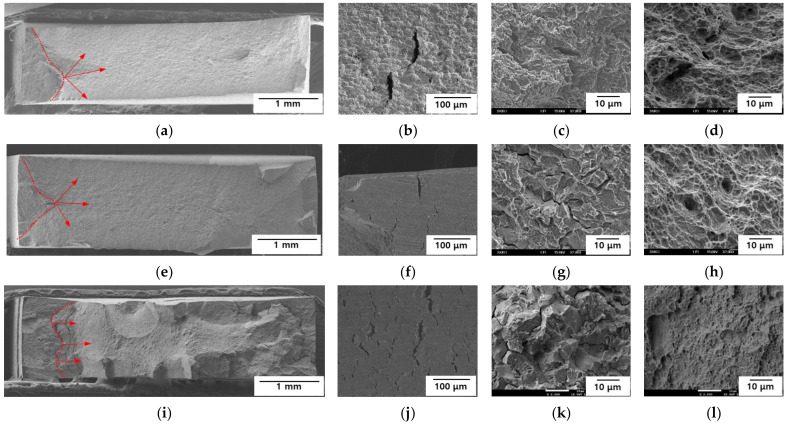
Fractography of Steel B at (**a**–**d**) −600 mV_SCE_, (**e**–**h**) −750 mV_SCE_, and (**i**–**l**) −1500 mV_SCE_. (**a**,**e**,**i**) Entire sample, (**b**,**f**,**j**) side view, (**c**,**g**,**k**) crack initiation site, and (**d**,**h**,**l**) center. Red arrows indicate the initiation of cracks and direction of propagation.

To determine the fracture properties of the AHSS samples, after the SSRTs were conducted, the fracture surfaces and sides of the specimens were observed by SEM. The results are presented in Figure 8 and Figure 9. Cracks were initiated and propagated from the sides in all the specimens. In Steel A, uniform pitting corrosion was observed on the sides at −600 mV_SCE_, while there was no changes at −750 and −1500 mV_SCE_ (Figure 8b,f,j). Dimples were observed at the crack initiation site at −600 mV_SCE_, cleavage occurred at the crack initiation site at −750 mV_SCE_, and transgranular fracturing was noted at −1500 mV_SCE_ (Figure 8c,g,k). All the specimens exhibited dimpling at the center of their fracture surfaces (Figure 8d,h,l). Steel A only exhibited ductile fracturing at −600 mV_SCE_, and the lower E_app_ values resulted in more brittle fracture behavior. Even under a potential of −1500 mV_SCE_, the center of the specimen exhibited ductile fracture behavior. Therefore, hydrogen did not diffuse into the center of the specimen.

In Steel B, uniform corrosion and cracks occurred on the side of the specimen at −600 mV_SCE_. Cracks without any corrosion were observed at −750 and −1500 mV_SCE_ (Figure 9b,f,j), and the lower E_app_ values were correlated with a higher density of cracks. Cleavage was observed at the crack initiation site at −600 mV_SCE_, while mixed intergranular and transgranular fractures were seen at the crack initiation sites of −750 and −1500 mV_SCE_ (Figure 9c,g,k). The intergranular fracture was more obvious at −1500 mV_SCE_, and in all the specimens, dimples occurred at the center of the fracture surfaces (Figure 9d,h,l). Steel B exhibited brittle fractures at −600 mV_SCE_, and the lower E_app_ values were associated with more obvious brittle fracture behaviors. At −1500 mV_SCE_, the center of Steel B demonstrated ductile fracture behavior. Thus, like Steel A, hydrogen did not diffuse into the center of the specimen. Under the same E_app_, Steel B exhibited more brittle fracture behavior than Steel A. The fractography results confirmed that, compared with Steel A, Steel B was more susceptible to delayed fractures.

### 3.4. Hydrogen Trapping and Desorption Behaviors

**Figure 10:**
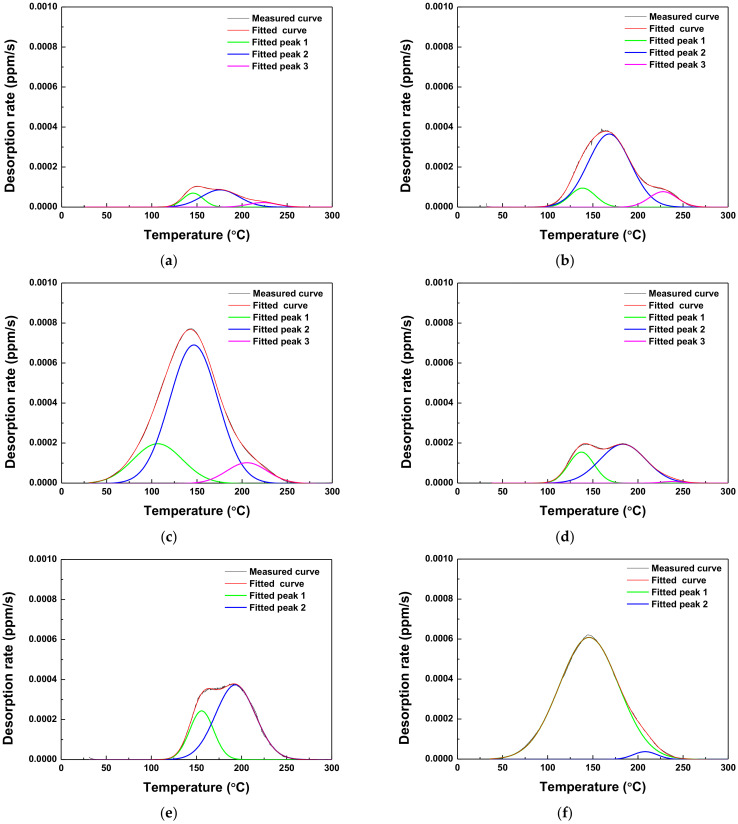

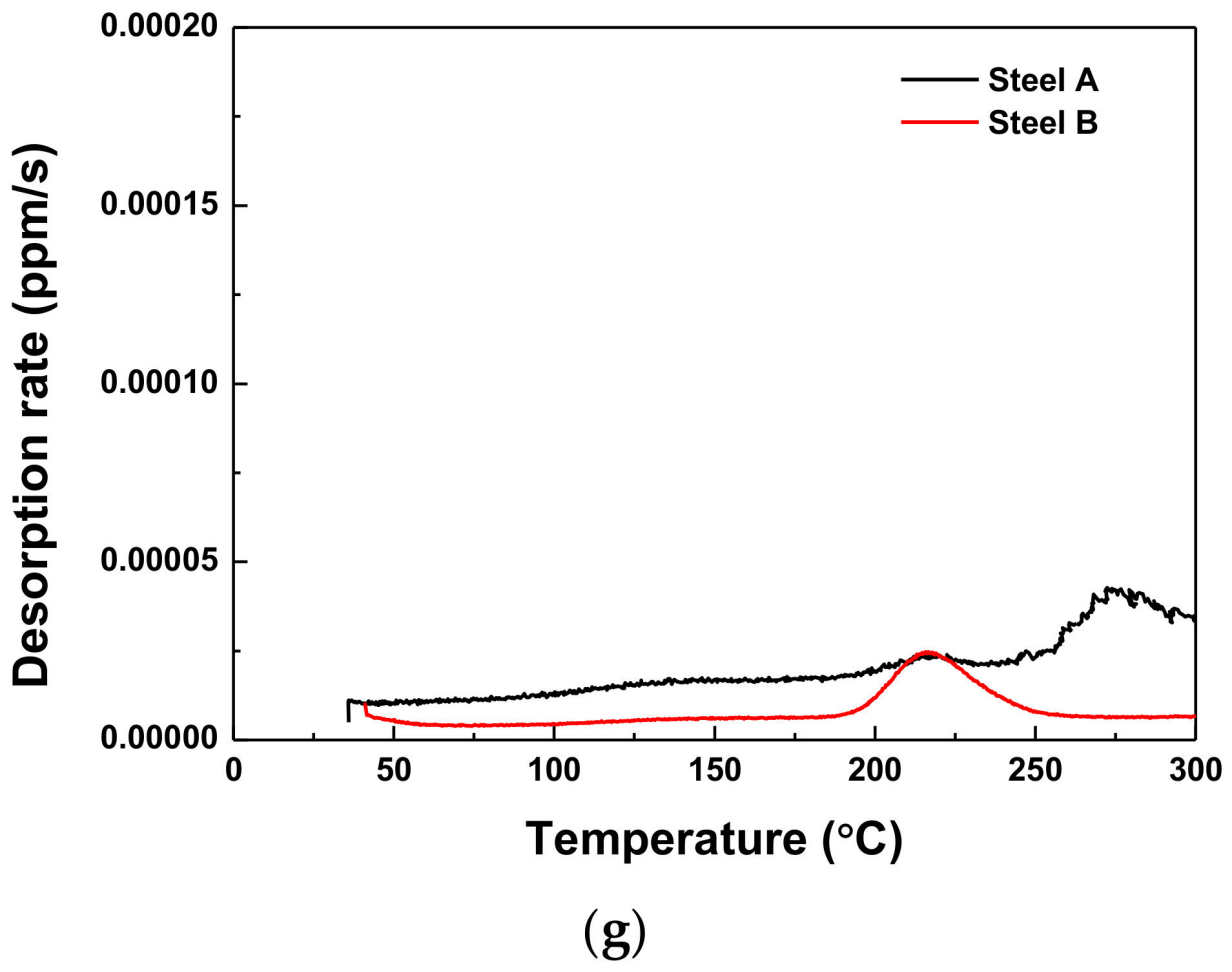
Hydrogen desorption rates obtained by thermal desorption spectroscopy at a heating rate of 4 °C/min in a fractured specimens of (**a**–**c**) Steel A and (**d**–**f**) Steel B at (**a**,**d**) −600, (**b**,**e**) −750, (**c**,**f**) −1500 mV_SCE_, and (**g**) the as-received condition.

To investigate the desorption behavior of diffusible hydrogen, the SSRT specimens were analyzed by TDS at the E_app_ values of −600, −750, and −1500 mV_SCE_. The results are presented in Figure 10 and Table 5. To quantitatively analyze the desorbed hydrogen, the area below the desorption rate vs. the temperature curve was integrated [19] (see Table 5 for the results). Just 0.05 ppm of diffusible hydrogen was released in the as-received specimens. Most of the diffusible hydrogen that accumulated during the steel manufacturing process (e.g., during acid cleaning) appeared to be released during machining and storage. However, when the potential was applied, the lower E_app_ was associated with the higher hydrogen desorption rate. Under the same E_app_ values of both AHSSs, the desorbed diffusible hydrogen content of Steel B was higher than in Steel A, except for −1500 mV_SCE_. In that case, Peak 3 of Steel A and Peak 2 of Steel B (located at approximately 220 °C) originated from the deformation field around the dislocation. In this study, as tensile deformation was considered an error, the hydrogen de-trapping from these peaks was negligible. Theoretically, the production of hydrogen did not occur at −600 mV_SCE_, although diffusible hydrogen was detected. It is assumed that the hydrogen was accumulated from the 1-h stabilizing process before the SSRTs were conducted.

To analyze the hydrogen-trapping sites in the steel specimens, the activation energy for hydrogen de-trapping was calculated using Equation (4), as proposed by Kissinger [20,21,22]:

(4) $$\frac{\partial \ln\left(\varphi /T_{c}^{2}\right)}{\partial \left(1/T_{c}\right)}=-\frac{E_{aT}}{R}$$

where *T_c_* is the temperature (K) at which the hydrogen desorption rate is maximal, *φ* is the heating rate (K/min), *E_aT_* is the activation energy for hydrogen de-trapping (kJ/mol), and *R* is the ideal gas constant (8.314 J/K∙mol).

**Figure 11:**
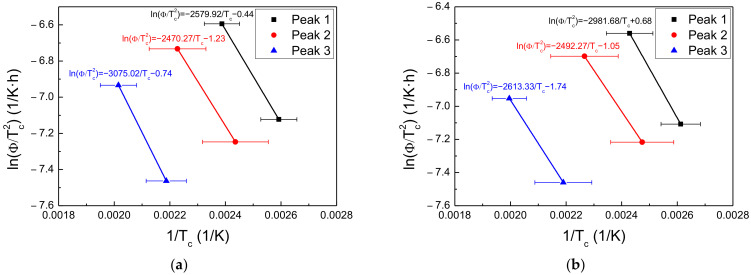

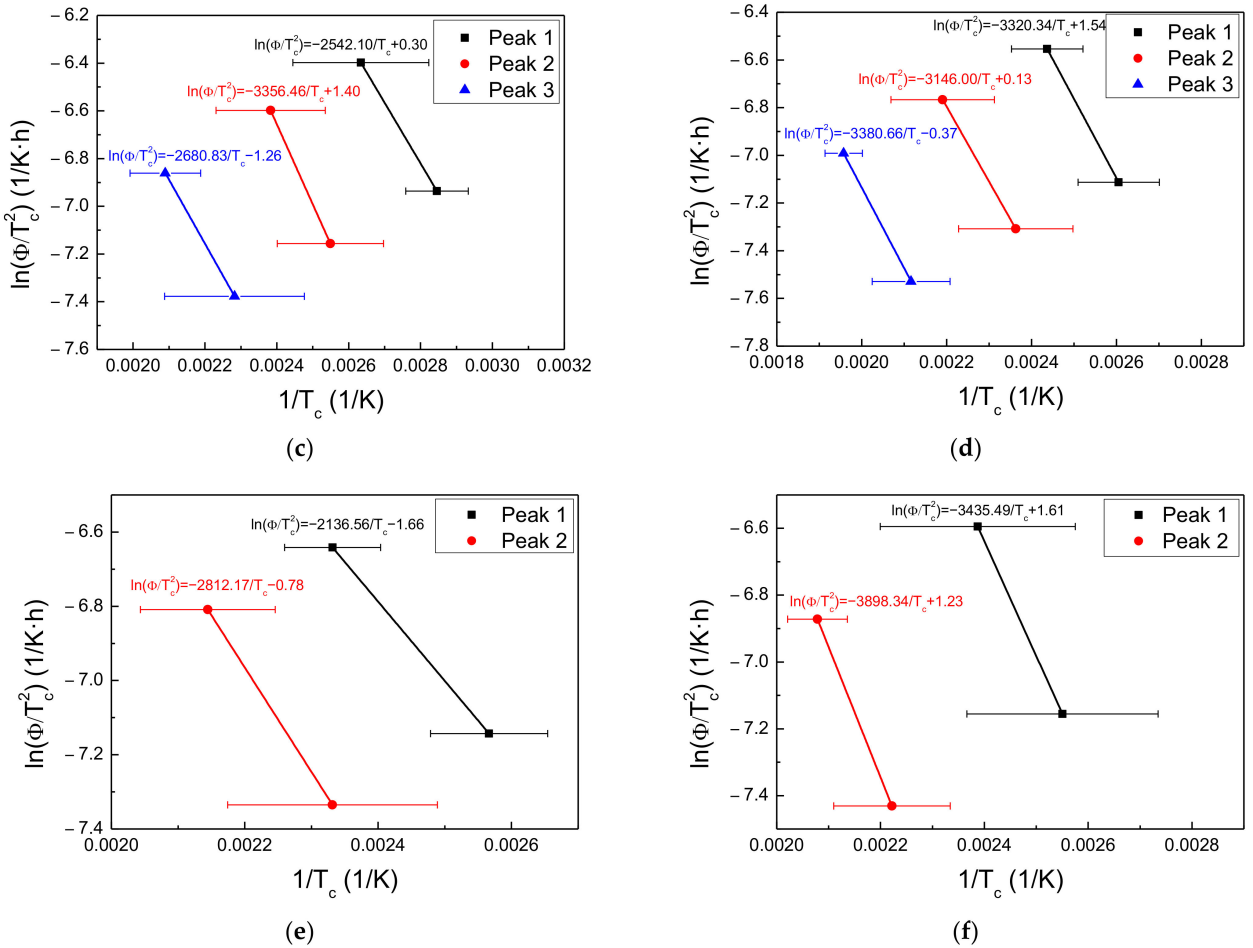
ln(*φ/T_c_*^2^) vs. 1/*T_c_* curve for (**a**–**c**) Steel A and (**d**–**f**) Steel B at (**a**,**d**) −600, (**b**,**e**) −750, and (**c**,**f**) −1500 mV_SCE_.

As is shown in Figure 10, the desorption curves were deconvoluted into two or three peaks of Gaussian curves, indicating that diffusible hydrogen accumulated at more than two or three trapping sites. According to the Kissinger equation, the slope of ln(φ/*T_c_*^2^) vs. 1/*T_c_* curve for each peak represents the activation energies (see Figure 11 for the results). The activation energies for Steels A and B corresponding to each peak are illustrated in Table 6.

Table 7 summarizes the activation energies for hydrogen de-trapping reported in previous related studies. Based on the published literature, the electrochemically accumulated hydrogen corresponding to Peaks 1 and 2 in Steel A was associated with the grain boundary and dislocation. Peak 3 was associated with the mechanical deformation by tensile deformation that occurred during the SSRTs [23]. For Steel B, the hydrogen corresponding to Peaks 1 and 2 at −600 and −750 mV_SCE_, respectively, was desorbed from the grain boundary, dislocation, and ferrite–Fe_3_C interface. In that specimen, the contributions from the grain boundary and dislocation were indistinguishable in Peak 1 at −1500 mV, which means that Peak 1 was the sum of the hydrogen desorbed from the grain boundary and dislocation. Peak 3 (−1500 mV_SCE_) was associated with mechanical deformation by tensile deformation, which occurred during the SSRTs.

### 3.5. Analysis of Defects Acting as Hydrogen Trapping Sites

#### 3.5.1. Electron Backscattered Diffraction Analysis

**Figure 12:**
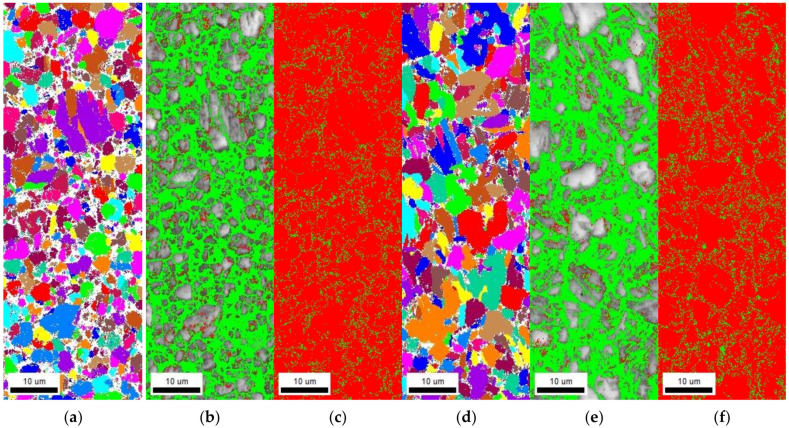
Electron backscattered diffraction results of (**a**–**c**) Steel A and (**d**–**f**) Steel B. (**a**,**d**) Grain mapping. (**b**,**e**) Low-angle grain boundary (red) and high-angle grain boundary (green) mapping. (**c**,**f**) Face-centered cubic (red) and body-centered cubic (green) mapping.

EBSD analysis was conducted to measure the grain boundary density and fraction of retained austenite; the results are shown in Figure 12 and Table 8. Each value was measured three times to derive the mean value. The average grain sizes measured by EBSD for Steel A and Steel B were 2.79 and 4.03 μm, respectively. Mild steel has an average approximate grain size of 22 μm [31]; therefore, these values indicate that AHSSs have a smaller grain size than mild steel.

When the misorientation of a grain boundary exceeds 15°, it is termed a high-angle grain boundary; otherwise, it is a low-angle grain boundary. In this study, Steel A had the longer high-angle grain length than Steel B, and the low-angle grain boundary lengths in both specimens were similar. The low-angle grain boundary is a reversible hydrogen-trapping site, suggesting that a longer low-angle grain boundary is more likely to induce HE [32]. Since the high-angle grain boundary is an irreversible hydrogen-trapping site, the longer high-angle grain boundary enhances the HE resistance. Therefore, in both AHSSs, the diffusible hydrogen content charged in the grain boundary is almost identical, and the non-diffusible hydrogen content charged in the grain boundary of Steel A is expected to be high.

The conducted EBSD analysis reveals that the face-centered cubic (FCC) structure reflected the retained austenite content. In Steel B, the retained austenite fraction was 10.9%, which was 1.4% higher than in Steel A. Retained austenite is an irreversible hydrogen-trapping site that enhances the HE resistance. However, in Steel B, the retained austenite fraction is not proportional to the HE resistance; this is because retained austenite with an FCC structure is transformed by tensile stress into martensite with a body-centered tetragonal (BCT) structure. Since BCT structures have a lower hydrogen solubility and faster diffusion rate than FCC structures, hydrogen accumulation via diffusion is easy in the BCT structure [33]. Thus, the hydrogen charged on the retained austenite in Steel B segregates during the tensile process and becomes susceptible to HE. Furthermore, the austenite–matrix interface is an effective diffusible hydrogen-trapping site [34]. The higher fraction of retained austenite increases the susceptibility to HE, i.e., it is expected that Steel B will be more susceptible to HE than Steel A.

#### 3.5.2. X-ray Diffraction

**Figure 13:**
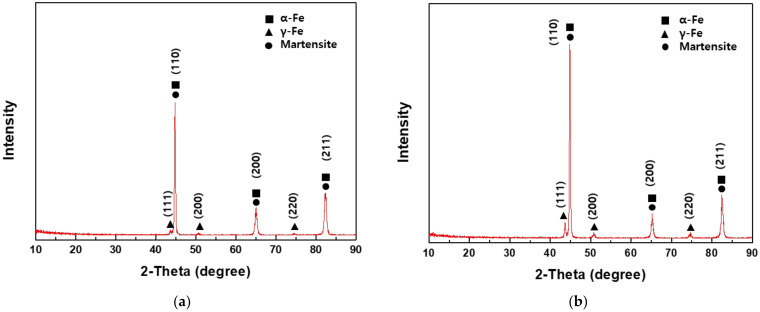
X-ray diffraction results for (**a**) Steel A and (**b**) Steel B.

The dislocation density of the samples was determined using XRD (see Figure 13 for the results). Both AHSSs were mainly comprised of α-Fe, although γ-Fe peaks were also observed. Specifically, the γ-Fe peaks were higher in intensity in Steel B compared with Steel A, which is consistent with the results of the EBSD analysis. The dislocation density is defined as the length of dislocation lines per unit volume of crystal and can be calculated using the Williamson–Smallman relationship [35], as in Equation (5) below:

(5) $$\delta =\frac{1}{D^{2}}$$

Here, *δ* is the dislocation density, and *D* is the size of crystalline domain, which is similar to the grain size. Therefore, *D* can be calculated using Scherrer’s equation [36], as follows:

(6) $$D=\frac{k\lambda }{\beta \cos\theta }$$

in which *k* is the shape factor (=approx. 0.9), *λ* is the wavelength (Cu-K_α_ = 1.5406 Å), *β* is the full width at half-maximum (FWHM) value, and *θ* is the position of the peaks. Using the above expression, the dislocation density was calculated to be 3.488 × 10^14^/m^2^ and 6.263 × 10^14^/m^2^ for Steel A and Steel B, respectively, i.e., the dislocation density of Steel B was twice that of Steel A. Since the low-angle grain boundary areas of the two AHSSs were similar, the difference in the hydrogen content of the two types of AHSSs discharged during TDS was attributed to the difference in the dislocation density.

#### 3.5.3. Characterization of Precipitates

**Figure 14:**
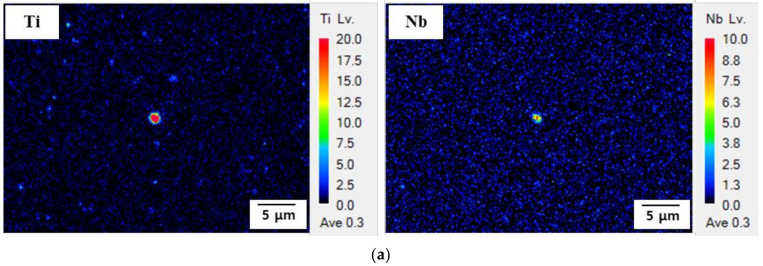

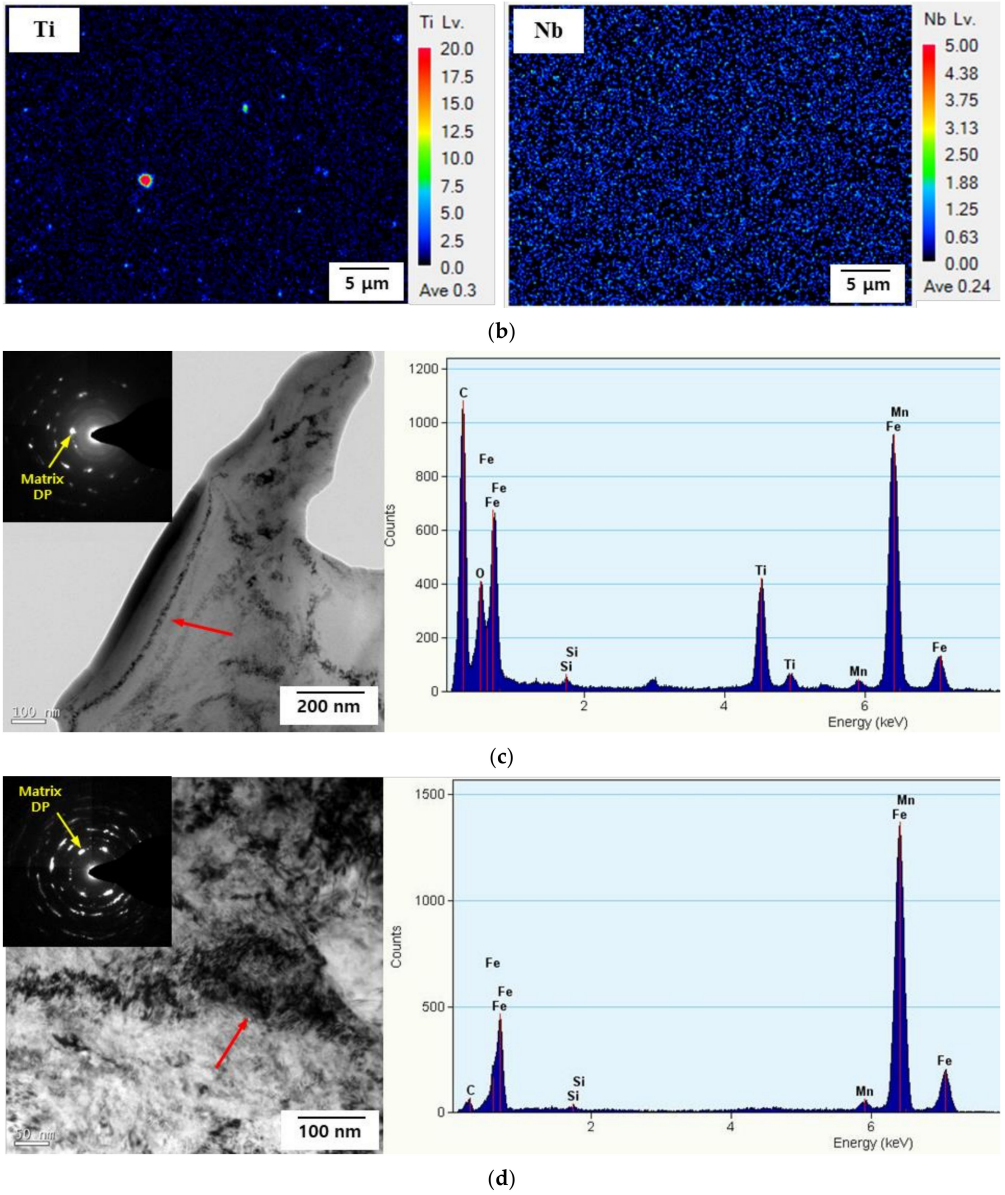
Identification of the types and sizes of precipitates on each AHSSs. Electron probe microanalysis results for (**a**) Steel A and (**b**) Steel B. Transmission electron microscopy results for (**c**) Steel A and (**d**) Steel B.

To characterize the type and size of the precipitates, EPMA and TEM analyses were conducted. The results are presented in Figure 14. According to Figure 14a, the precipitate of Steel A was rich in Ti and Nb and a (Nb, Ti) precipitate surrounded the Ti-rich precipitate. However, in the precipitates of Steel B, only Ti was detected, while Nb was undetected. The precipitates of both AHSSs were approximately 1 μm in size. The TEM images and diffraction patterns for the two types of AHSSs are presented in Figure 14c,d. In Steel A, extremely fine precipitates were distributed along the grain boundary. The electron diffraction pattern and energy-dispersive X-ray spectroscopy analysis confirmed that the precipitates were amorphous Ti and Fe carbides smaller than 10 nm in size. Only the small fraction of Fe carbides was distributed randomly in the grain, and in Steel B, no TiC precipitate was observed (Figure 14d). The EPMA and TEM results revealed the presence of sub-micrometer (Nb, Ti)C and fine TiC precipitates in Steel A, although Steel B contained only a sub-micrometer TiC precipitate. The small size of the carbide produced a large effective area for hydrogen trapping [37,38]. Therefore, Steel A was able to trap considerably more hydrogen in the TiC precipitate interface compared with Steel B. Since Nb and Ti precipitates are powerful and irreversible hydrogen-trapping sites, they can positively influence HE resistance, i.e., Steel A is expected to be more resistant to HE than Steel B.

## 4. Conclusions

This study investigated the SCC and HE mechanisms of two AHSSs using SSRTs and characterized their hydrogen-trapping behaviors using TDS, EBSD, and XRD. According to the results of these investigations, the SCC and HE characteristics of the studied AHSSs can be summarized as follows:

1. For both AHSSs, elongation decreased as the cathodic overvoltage increased, i.e., both types of AHSSs were fractured by the mechanism of HE. Even when the anodic potential was applied, HE was more dominant than SCC. Although the HE sensitivity of Steel B was higher than that of Steel A, both AHSSs were more sensitive to HE than SCC.
2. In both AHSSs, the lower E_app_ was associated with a strong brittle fracture behavior. However, the center of each specimen exhibited ductile fracture behavior, because the hydrogen did not diffuse into that region. It was clear that the fracture surface of Steel B was more brittle than that of Steel A.
3. The lower E_app_ was associated with the higher rate of hydrogen desorption. In both AHSSs, diffusible hydrogen was trapped mainly at the grain boundary and dislocation.
4. The density of the irreversible hydrogen-trapping sites (high-angle grain boundaries and TiC precipitates) was higher in Steel A than in Steel B. However, the density of the reversible hydrogen-trapping sites (low-angle grain boundaries and dislocations) was lower in Steel A than in Steel B. Therefore, compared to Steel A, Steel B was more susceptible to HE.

## Figures and Tables

**Table 1:** Chemical compositions of the advanced high-strength steels for use in automobiles (wt.%).

Component	C	Si	Mn	Cr	Ti	Nb	Fe
Steel A	0.11~0.18	0.4~0.7	2.2~2.7	0.0~0.1	0.01~0.02	0.01~0.02	Bal.
Steel B	0.11~0.18	1.2~1.9	2.4~2.7	0.20~0.45	0.01~0.02	-	Bal.

**Table 2:** Chemical composition (wt.%) of the modified Society of Automotive Engineers’ solution.

NaCl	CaCl_2_	NaHCO_3_	(NH_4_)_2_SO_4_	pH
0.5	0.1	0.075	0.35	7.3

**Table 3:** Potentiodynamic polarization test results.

Specimen	E_corr_ (V_SCE_)	I_corr_ (A/cm^2^)	β_a_ (mV/Decade)	β_c_ (mV/Decade)	Corrosion Rate (mm/y)
Steel A	−0.748	3.54 × 10^−5^	92	205	0.41
Steel B	−0.755	3.38 × 10^−5^	61	241	0.39
Mild steel	−0.765	1.70 × 10^−5^	89	419	0.20

**Table 4:** Slow strain rate test results.

Steel	Applied Potential (mV_SCE_)	Yield Strength (MPa)	Tensile Strength (MPa)	Elongation (%)	HE Susceptibility Index, I_HE_ (%)	Hydrogen Evolution Rate (g)
Steel A	Air	781	1186	16.0	-	-
	−600	703	1124	14.1	11.9	-
	−750 (E_corr_)	699	1146	11.2	30.1	5.76 × 10^−6^
	−1500	690	1041	4.6	71.0	1.23 × 10^−3^
Steel B	Air	800	1202	21.6	-	-
	−600	906	1150	16.4	24.0	-
	−750 (E_corr_)	852	1119	13.3	38.3	2.62 × 10^−6^
	−1500	851	1034	3.7	82.9	9.76 × 10^−4^

**Table 5:** Desorbed hydrogen contents for each peak.

Steel	Applied Potential (mV_SCE_)	Peak 1 (ppm)	Peak 2 (ppm)	Peak 3 (ppm)	Sum of Peaks (ppm)
Steel A	As-received	0.0569	-	-	0.0569 ± 0.0323
	−600	0.0622	0.1511	0.0311	0.2485 ± 0.1262
	−750	0.0579	0.3693	0.0514	0.4787 ± 0.0145
	−1500	0.2264	0.7735	0.0978	1.0977 ± 0.0968
Steel B	As-received	0.0506	-	-	0.0506 ± 0.0268
	−600	0.1388	0.3122	0.0057	0.4568 ± 0.2070
	−750	0.1748	0.4445	-	0.6193 ± 0.1280
	−1500	0.9622	0.0246	-	0.9868 ± 0.0052

**Table 6:** Calculated activation energies for hydrogen de-trapping.

Steel	Applied Potential (mV_SCE_)	Peak 1 (kJ/mol)	Peak 2 (kJ/mol)	Peak 3 (kJ/mol)
Steel A	−600	21.5	20.5	25.6
	−750	24.8	20.7	21.7
	−1500	21.1	27.9	22.3
Steel B	−600	27.6	26.1	28.1
	−750	17.8	23.4	-
	−1500	28.6	32.4	-

**Table 7:** Types of reversible and irreversible hydrogen-trapping sites reported in the literature.

Type of Trap	Activation Energy (kJ/mol)	References
**Reversible hydrogen-trapping sites**		
Ferrite/Fe_3_C	10.9	[16]
Grain boundary	17.2	[16]
Ferrite/Fe_3_C interface	18.4	[16,24]
Grain boundary, Dislocation	21–29	[25,26,27,28]
Deformation field around dislocation	29 ± 5	[23]
**Irreversible hydrogen-trapping sites**		
Semi-coherent TiC	49.9	[28]
High-angle grain boundary	53–59	[29]
NbC interface	63–68	[30]
Incoherent TiC	85.7, 86.9	[28]

**Table 8:** EBSD analysis results (relative value).

Specimen	Average Grain Size	High-Angle Grain Boundary Length	Low-Angle Grain Boundary Length	Retained Austenite Fraction
Steel A	2.79 μm	15.73 mm	1.91 mm	9.5%
Steel B	4.03 μm	13.93 mm	1.99 mm	10.9%

## Data Availability

Not applicable.
